# Beyond the Tradition in Tympanoplasty: A Retrospective Cohort Study of a New Modified Palisade Tympanoplasty Technique

**DOI:** 10.7759/cureus.114479

**Published:** 2026-08-13

**Authors:** Rula Naqi, Israa Sinan, Noora Althawadi, Hesham Alrayyes

**Affiliations:** 1 Otolaryngology - Head and Neck Surgery, King Hamad University Hospital, Royal Medical Services, Muharraq, BHR; 2 School of Kinesiology and Health Science, York University, Toronto, CAN; 3 Otolaryngology, Northwick Park Hospital, London, GBR

**Keywords:** hearing loss, modified palisade tympanoplasty, otology, retrospective studies, tympanic membrane perforation, tympanoplasty

## Abstract

Background & aim: Tympanic membrane perforation is a common cause of conductive hearing loss and otologic morbidities. The most common cause is trauma, which tends to heal spontaneously. Conversely, perforations caused by infections or middle ear pathology require surgical intervention, such as tympanoplasty. While cartilage tympanoplasty provides benefits of durability and resistance to retraction, concerns persist regarding graft rigidity and its adverse effects on hearing outcomes. This study was implemented to assess the anatomical and functional success of an innovative and novel form of modified palisade cartilage tympanoplasty, which is designed to improve graft flexibility, stability, and audiological results.

Materials & methods: A retrospective cohort study was conducted at a tertiary referral center between 2019 and 2023. Nineteen patients (age range = 9-64 years) who underwent the modified palisade tympanoplasty using a tragal composite cartilage-perichondrium graft were included, and alternative grafting techniques were excluded. Variables including demographic data, clinical history, surgical information, and postoperative follow-up regarding graft uptake and both functional and anatomical success were assessed for each patient. Functional success was defined as a postoperative air-bone gap (ABG) ≤ 20 dB, while anatomical success was defined as an intact, dry tympanic membrane. Pre- and postoperative audiometric outcomes were compared using paired statistical analysis.

Results: Complete graft uptake was accomplished in 89.5% (N = 17) of cases. Anatomical success was achieved in 78.9% (N = 15) of patients, and functional success in 84.2% (N = 16). Audiometric evaluation showed a statistically significant improvement in postoperative ABG compared to preoperative values (p = 0.004). Disrupted ossicular chain and the presence of cholesteatoma or tympanosclerosis were significantly associated with poorer outcomes (p < 0.001). No significant associations were found between outcomes and patient demographics, perforation characteristics, smoking status, or surgical approach. Favorable results were achieved across surgeons with varying levels of experience.

Conclusions: The modified palisade tympanoplasty technique is shown to have effective graft uptake and considerable improvement in hearing by overcoming the stiffness of the cartilage graft. The effectiveness of this technique has been exhibited across a wide age range, co-existing health conditions, and different levels of surgeon expertise, proving that all patients can be subjected to the procedure. Further studies need to be conducted to validate our findings.

## Introduction

Hearing loss is a common medical condition that increases in incidence and severity with age [1]. Modern practice has allowed for the diagnosis and management of a wide range of hearing loss pathologies in both children and adult populations [2]. One of the most common causes of conductive hearing loss is a perforated tympanic membrane, of which 10-15% occurs following trauma [3]. Approximately 80-90% of traumatic tympanic membrane perforations heal spontaneously without any modality of surgical intervention [4]. On the other hand, around 84% of tympanic membrane perforations are caused by infections, including acute and chronic suppurative otitis media [5]. Failure to heal might impose consequences and cause morbidity to the patient in the form of conductive hearing loss or recurrent ear infection [6]. In such cases, surgical intervention to repair the tympanic membrane perforation, such as tympanoplasty, is warranted [7]. Different techniques, approaches, and graft materials have been developed and proposed over the years for tympanoplasties [8]. More recently, cartilage tympanoplasty has gained confidence in terms of ease and reliability in outcomes [9]. However, it has a disadvantage of reduced vibratory function of the tympanic membrane, which can worsen hearing outcomes [10]. Thus, palisade tympanoplasty has been established [11,12]. In this technique, the cartilage is reshaped and not inserted as a single piece. The aim is to improve mobility, reduce acoustic impedance, and improve hearing outcomes [13]. Furthermore, there is still some controversy in the outcome of this technique, as some studies showed excellent outcomes while others showed outcomes similar to conventional cartilage tympanoplasty [12,14].

This retrospective study aims to observe the efficacy of an innovative modified palisade tympanoplasty technique by assessing postoperative outcomes. It includes both subjective outcomes, such as the history of otorrhea and recurrent ear infections, and objective outcomes, which consist of assessing graft uptake, the presence of any residual perforation, and audiometric assessment.

## Materials and methods

Study design

This is a retrospective cohort study to assess the efficacy of the innovative modified palisade tympanoplasty technique in improving postoperative subjective and measured outcomes in patients who have tympanic perforation.

Sampling method

Data were collected from the electronic medical records of King Hamad University Hospital (KHUH), a tertiary medical center in the Kingdom of Bahrain. All patients who underwent the modified palisade tympanoplasty were included in the study from 2019 to 2023. Patients who underwent paper myringoplasty, tympanoplasty using temporalis fascia, or any other conventional cartilage grafting techniques were excluded.

Data collection

Demographic data collected included age, gender, smoking status, and comorbidities, as well as whether there was any history of tympanoplasty surgery. Surgical data included the indication, date of surgery, duration of preoperative dry ear status, surgical approach (transcanal or postauricular), type of dressing applied, and operating surgeon (consultant or senior). Perforation-related data included the side of the ear, site, size, etiology, status at the time of presentation, status of the other ear, mucosal lining of the middle ear, and ossicular chain condition. Postoperative data included a history of otorrhea, infections, complications, and graft uptake. Moreover, functional success, defined as an air-bone gap ≤20 dB on postoperative audiometry, and anatomical success, defined as maintaining an intact graft with a dry ear, were assessed.

Ethical approval

This study was approved by the Institutional Review Board at our institution. The reference number is IRB# 26-608. It was obtained on 17 April 2023. All data were collected without any identifying information to ensure the confidentiality and privacy of patients.

Modified surgical technique of palisade tympanoplasty

The initial steps of the modified palisade tympanoplasty are similar to those of traditional tympanoplasty surgical techniques. Edges of the perforation were freshened along with scratching the surrounding inner surface of the tympanic membrane, and a tympanomeatal flap was raised from 2 o’clock up to 11 o’clock, if needed. A transcanal approach was mostly used with no flap raised. A trimmed thin silastic sheet was used to size the perforation and determine the optimum graft size required to fit the perforation.

**Figure 1:**
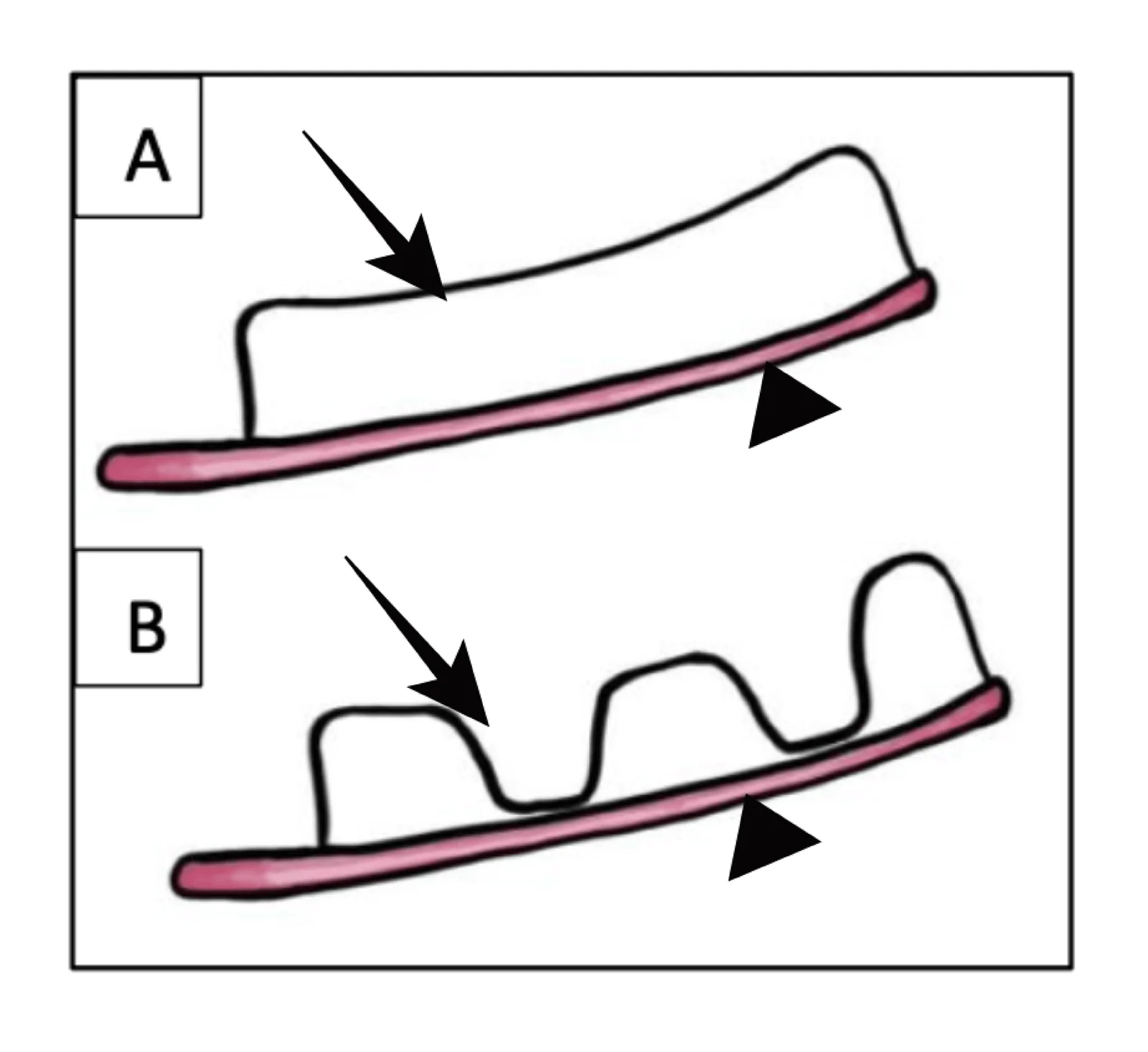
(A) Schematic diagram of the harvested composite graft showing cartilage (white) with an intact underlying layer of perichondrium (pink). (B) Harvested graft after undergoing non-through-through longitudinal excision of strips of cartilage while maintaining the underlying layer of perichondrium. Original schematic diagram. The arrow indicates the perichondrium, and the arrowhead indicates the cartilage.

A dorsal incision at the free margin of the tragus and a tragal composite graft was obtained by dissecting the tragal cartilage and its adherent perichondrium from the skin flaps anteriorly and posteriorly. The graft was harvested slightly larger than the size of the perforation, as gauged by the size of the reference silastic sheet, while a free rim of the dissected cartilage was spared untouched to maintain the cosmetic contour of the tragus. One side of the harvested cartilage was skeletonized and freed from the attached perichondrium. The cartilage graft was then trimmed down in size to be slightly bigger than the size of the perforation. The sized cartilage was left attached centrally to the free perichondrium, from which the excess cartilage was removed, and this served to ensure stability of the cartilage via an underlay technique as well as improved graft uptake (Figure 1A).

**Figure 2:**
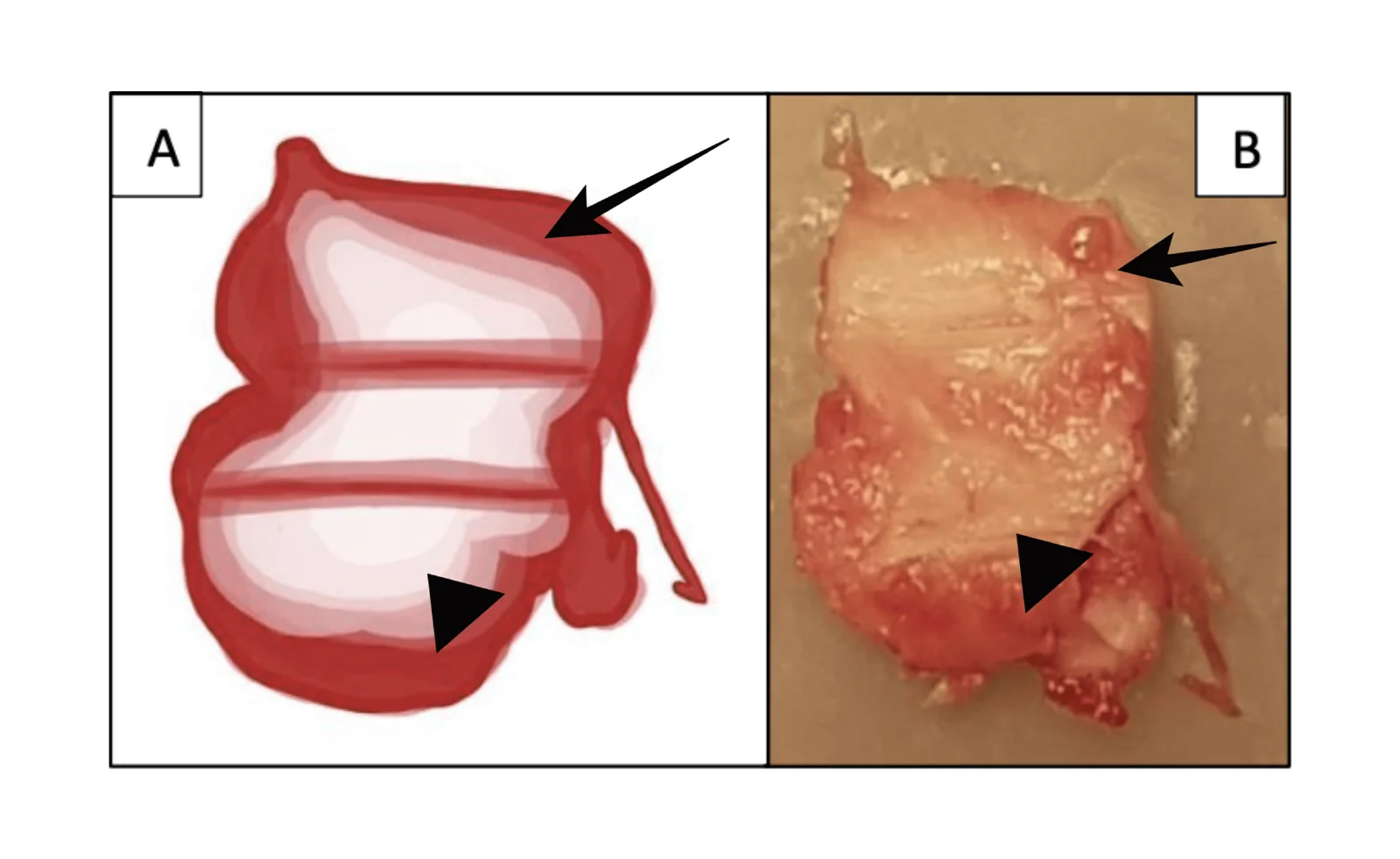
Top view of the harvested graft following the modified palisade technique. (A) Schematic representation. (B) Graft used in the case described. Original schematic diagram. The arrow indicates the perichondrium, and the arrowhead indicates the cartilage.

The cartilaginous island graft obtained was subjected to a partial non-through-through longitudinal wedged-strip excision, ensuring that no excised strips are left adherent to the underlying perichondrium, thus acting like palisades held together by underlying perichondrium (Figures 1B, 2).

Finally, the attached perichondrium was withdrawn from underneath the tympanomeatal flap to stabilize the attached cartilage in the intended place as an underlay technique. For large or medium-sized perforations, the graft was inserted through the perforation itself and supported medially with Gelfoam. The attached perichondrium helped stabilize the graft and promote uptake by healing to the scarified medial aspect of the tympanic membrane. The cartilage and the tympanomeatal flap were then covered with Gelfoam soaked in antibiotic ointment to support the healing graft.

## Results

Sociodemographic data of the participants

Data analysis was conducted using SPSS v.25 (IBM Corp., Armonk, NY). A p-value of less than 0.05 was considered significant. A total of 19 patients were included in the study, representing all cases that underwent the modified palisade tympanoplasty technique since 2019 at KHUH. The surgery was done by the primary consultant who pioneered the technique. The average age of the participants was 36.6 ± 19.1 years, with a minimum age of nine years and a maximum age of 64 years. Most of the patients were female (78.9%) (N = 15). Out of all cases, there were three pediatric cases (21%), with ages ranging from nine to 16 years, who were all female. One of them underwent tympanoplasty in both ears. The most common comorbidity was dyslipidemia (26.32%) (N = 5) (Table 1).

**Table 1:** Population demographics.

Variable	Descriptive statistics (n = 19)	
Age, mean ± SD (range)	36.6 ± 19.1 (9-64)	
Gender, n (%)	Male	4 (21.05%)
	Female	15 (78.95%)
Comorbidities, n (%)	Diabetes mellitus	4 (21.05%)
	Asthma	2 (0.53%)
	Dyslipidemia	5 (26.32%)
	Hypertension	4 (21.05%)
	Hyperuricemia	1 (5.26%)
	Hypothyroid	2 (10.53%)
	Psoriasis	1 (5.26%)
Smoking history, n (%)	Yes	2 (10.53%)
	No	17 (89.47%)

Preoperative data

Clinical signs and symptoms of the patients were noted (Table 2).

**Table 2:** Preoperative clinical status of the population.

Variable	Clinical status	Descriptive statistics (n = 19)
Side of tympanic membrane perforation, n (%)	Left ear	10 (52.6%)
	Right ear	9 (47.4%)
Symptoms, n (%)	Recurrent infections	13 (68.4%)
	Hearing loss	10 (52.6%)
	Otorrhea	5 (26.3%)
	Tinnitus	2 (10.5%)
	Otalgia	2 (10.5%)
Previous tympanoplasty, n (%)	Yes	3 (15.8%)
	No	16 (84.2%)
Site of perforation in the tympanic membrane, n (%)	Anterior	2 (10.5%)
	Central	5 (26.3%)
	Posterior	6 (31.6%)
Perforation size, n (%)	Small perforation (less than 25%)	2 (10.5%)
	Medium perforation (25 to 50%)	2 (10.5%)
	Large perforation (over 50% and subtotal)	9 (47.4%)
Etiology of perforation, n (%)	Trauma	1 (5.3%)
	Infection	3 (15.8%)
	Iatrogenic	3 (15.8%)
Status of opposite ear, n (%)	Normal	13 (68.4%)
	Perforated	2 (10.5%)
	Repaired perforation	4 (21.1%)

All patients had a dry perforation preoperatively for a duration of three months, as a standard practice for the procedure.

Postoperative data

**Figure 3:**
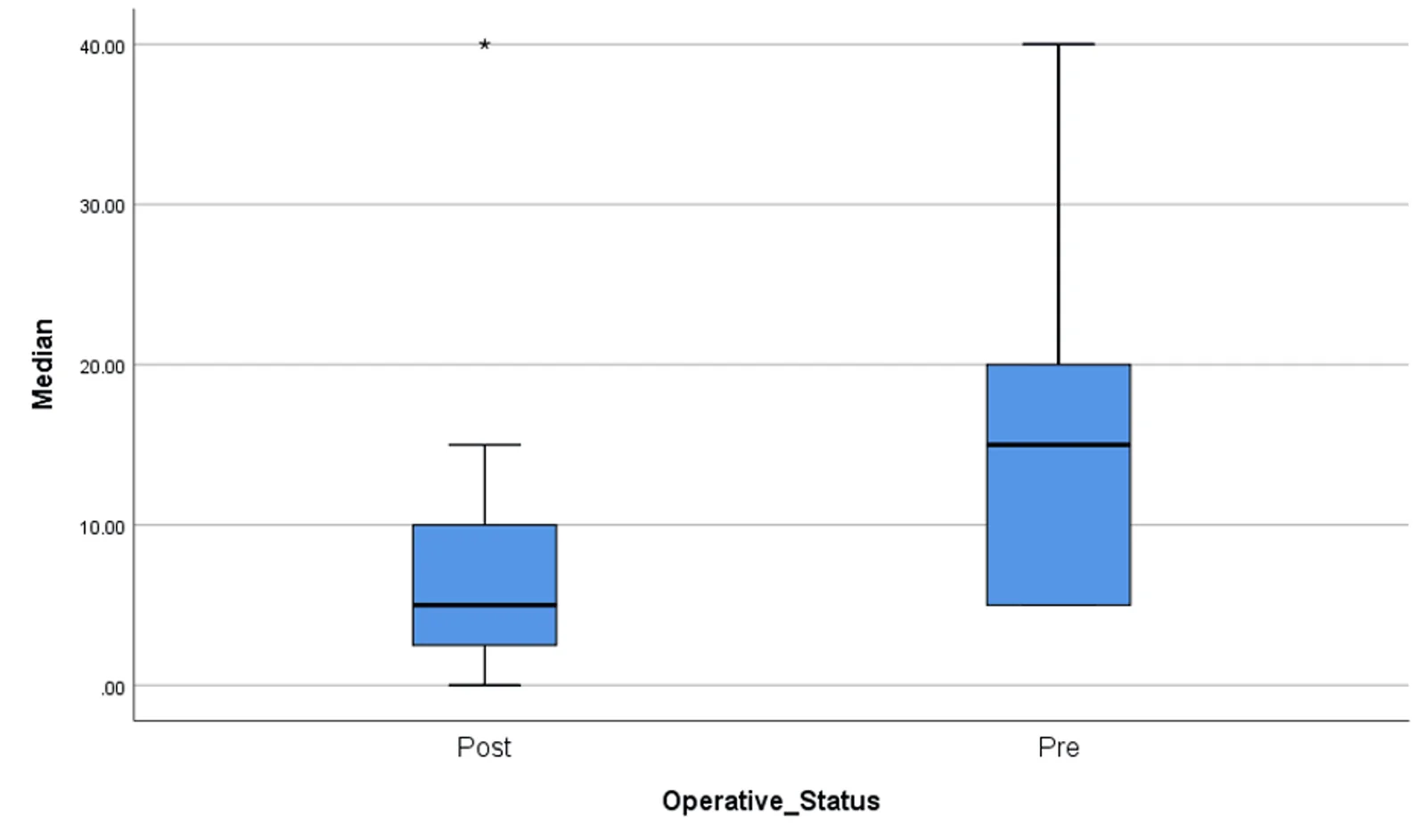
Box plot comparing the median levels of air-bone gap for all patients pre- and postoperatively. * Outlier in the group of postoperative patients.

Audiometry was conducted preoperatively and postoperatively, and the median air-bone gaps (in decibels) were assessed at the following frequencies: 250, 500, 1,000, 2,000, and 4,000 Hz. This was used to showcase the changes that the technique had on the air-bone gap, which represents the state of the perforation in terms of size and hearing level. A paired t-test showed that these changes were significant (p = 0.004) (Figure 3).

**Figure 4:**
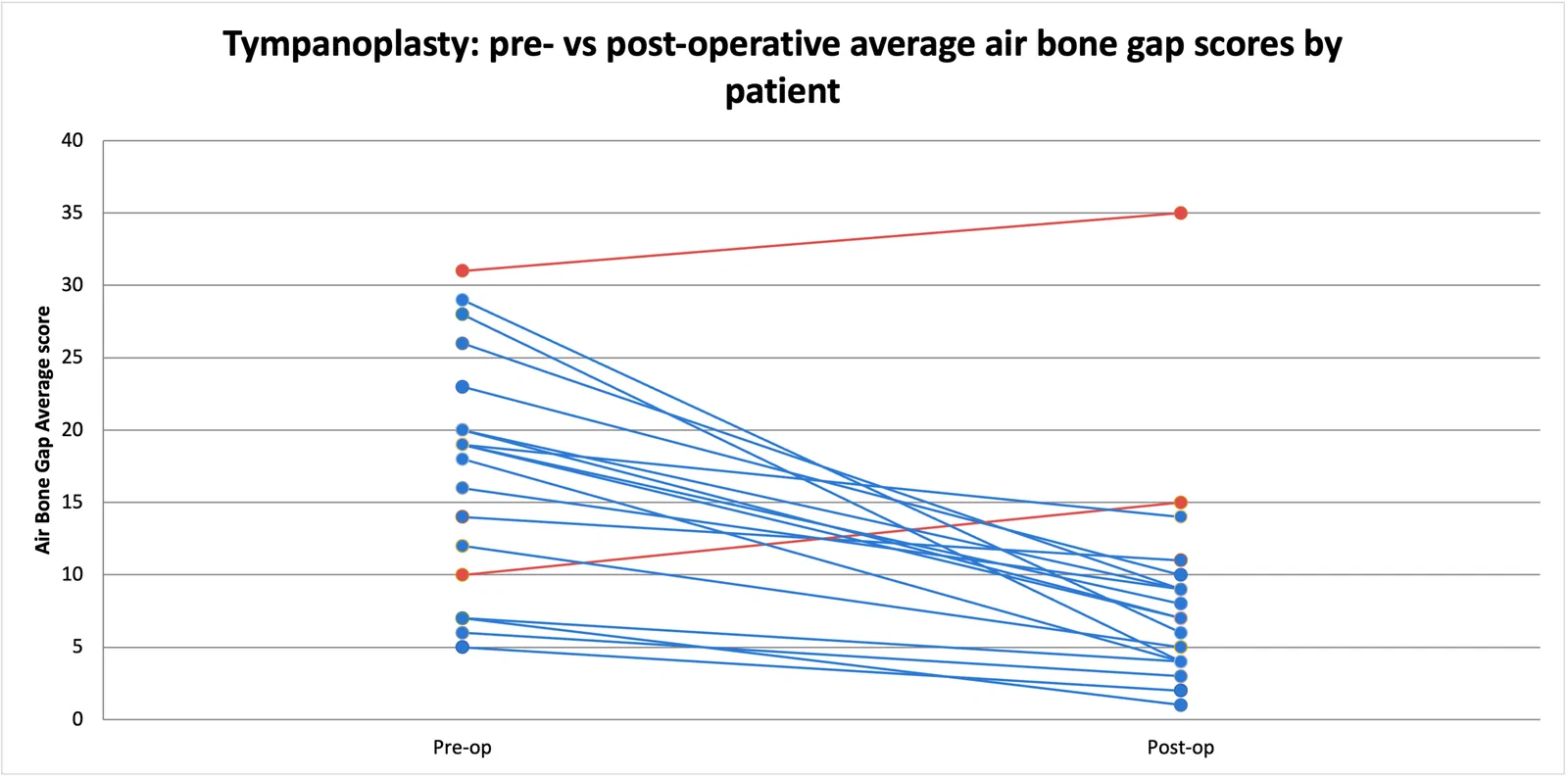
Comparing the mean average of air-bone gap for each patient pre- and postoperatively.

Means were also calculated, and they showed the changes that the technique had on operative status (Figure 4).

It is predicted that if anatomical success is achieved, then functional success has also been achieved. Based on this, the overall positive success rate of this technique was 89.4% (N = 15), and the true positive success rate was 68.4% (N = 13). The positive predicted value for this procedure was calculated to be 76.4% (Table 3).

**Table 3:** Overall success based on predicted anatomical success and actual functional success of the surgery (n = 19).

	Functional success		
Anatomical success, n (%)		Achieved	Not achieved
	Achieved	13 (68.4%)	2 (10.5%)
	Not achieved	4 (21.0%)	0 (0.0%)

Some patients had a disrupted ossicular chain, either tympanosclerosis or cholesteatoma, which had a significant impact on the overall successful outcome (<0.001). There were around 10.5-15.8% (N = 2-3) complications postoperatively, which were otorrhea and otitis media, respectively. As for graft uptake, it was 89.5% (N = 17), with only two cases having perforation. Intra- and postoperative clinical status of the population is presented in Table 4.

**Table 4:** Intra- and postoperative clinical status of the population.

Variable	Clinical status	Descriptive statistics (n = 19)
Middle ear mucosal status, n (%)	Normal	15 (79.0%)
	Cholesteatoma	3 (15.7%)
	Tympanosclerosis	1 (5.3%)
Ossicular chain status, n (%)	Intact	14 (73.7%)
	Disrupted	5 (26.3%)
Approach, n (%)	Post-auricular	3 (15.8%)
	Transcanal	16 (84.2%)
Postoperative otorrhea, n (%)	Yes	2 (10.5%)
	No	17 (89.5%)
Postoperative infections, n (%)	Yes	3 (15.8%)
	No	16 (84.2%)
Graft take, n (%)	Complete	17 (89.5%)
	Incomplete	2 (10.5%)
Perforation, n (%)	Yes	2 (10.5%)
	No	17 (89.5%)
Functional success, n (%)	Yes	16 (84.2%)
	No	3 (15.8%)
Anatomical success, n (%)	Yes	15 (78.9%)
	No	4 (21.1%)

The operating surgeon in 14 cases was a consultant, who had over 20 years of experience, and the operating surgeons in five other cases were senior residents who had less than 10 years of experience. The operating consultant had a 78.5% (N = 18) positive success rate, and the senior residents had a 60% (N = 17) positive success rate. Neither of the operating surgeons had a truly negative outcome, indicating that this technique can be utilized under a number of conditions with proper training.

## Discussion

Tympanoplasty is a type of surgery that involves repairing the perforated tympanic membrane with ossiculoplasty [7]. It is divided into five types, depending on the extent of disease involvement in the ossicular chain [7].

Different materials have been used as a graft in tympanoplasty [7,15,16], including autologous grafts, which include temporalis fascia, perichondrium, cartilage, vein, fat, and periosteum, and alloplastic grafts, which include acellular dermal matrix and absorbable gelatin sponge [7,15]. Although alloplastic grafts avoid morbidities related to harvesting a graft, they are associated with a higher risk of infections along with higher cost [8,16]. Thus, surgeons usually avoid using them, preferring autologous grafts [7,16].

The most common type of graft initially used was temporalis fascia, with a success rate between 93% and 97% in the primary form of tympanoplasty [16]. However, in revision surgeries, the best option was cartilage grafts [15]. Recently, interest has been raised in using cartilage grafts directly due to observations of high strength and stability, making it more resistant to shrinkage [7,16]. Moreover, cartilage tympanoplasty is highly accepted because the most common indication is chronic secretory otitis media, representing around 93% of all cases [17]. However, this fact has made the choice of cartilage tympanoplasty debatable, as resistance to shrinkage indicates stiffness, which might affect hearing negatively [10]. Further studies have been conducted to resolve this debate, supporting the hearing outcomes of cartilage tympanoplasty [10].

A palisade tympanoplasty technique has been established [11]. The cartilage graft is reshaped into a palisade of 0.5-3 mm thickness, which is placed alongside each other in an overlapping manner [11,12]. The aim is to improve mobility, reduce the acoustic impedance, and improve hearing outcomes. Furthermore, there is controversy in outcomes, as some studies demonstrated excellent results of this technique, while others showed no difference between it and using the cartilage graft as a single piece [12,14].

In our new tympanoplasty technique, where the tragal cartilage has been used, the air-bone gap was statistically and clinically significantly improved (Figures 3, 4). This could be because, in this modified technique, the cartilage graft underwent non-through-through longitudinal strip excision. This is contrary to the known palisade tympanoplasty, where longitudinal strips of cartilage are separated from the perichondrium on both sides [12]. This step is crucial as it allows flexibility of the cartilage during insertion. This modified palisade technique further aids the spread of the cartilage once properly positioned in the perforation and enables better sealing of the perforation in a fashion similar to the musical accordion. Additionally, this step ensured elimination of the stiffness of the cartilage as well as rigidity that could impose trauma to the remnant tympanic membrane and ossicles during insertion and avoid the negative impact on hearing outcomes [18].

Upon comparing our study results with studies on the palisade tympanoplasty technique, we found similar outcomes. Arora et al. [11] had a graft uptake of 90%, with failure in 10% and a mean postoperative air-bone gap closure of 8.7 dB. Moreover, Khan et al. [19] had 97.8% graft uptake with 10% complications. The complications were similar to our study, which were perforation, recurrence, and otorrhea. However, Vashishth et al. [20] had 90% graft uptake and 0% complications. Demirpehlivan et al. [21] had 79% graft uptake and 10% complications, similar to our study. Having similar outcomes in various studies conducted in different parts of the world is considered a positive finding, as it indicates that such techniques are excellent and highly applicable. Having further studies with a larger number of patients will help in determining any further clinical or statistical differences.

Likewise, as shown by Ciğer et al. [22] and Guler et al. [23], cartilage graft has been proven to be superior to temporalis fascia grafts in tympanoplasty outcomes across a wide age group. The stiffness of cartilage can pose a challenge in graft material insertion, whether underlay or transperforation. Our technique overcomes this limitation by providing the cartilage with some maneuverability and resilience, yet maintaining its integrity. This additional layer of perichondrium further contributes to the stability of the graft and helps establish blood supply to the cartilage, thus raising tympanoplasty success rates [12]. Another factor contributing to graft uptake is the surface area of the graft. Increasing the surface area using the above-described modified palisade technique further contributes to improved healing and likelihood of tympanoplasty success. This is comparable to increasing the success rate by meshing a split-thickness skin graft [24].

Furthermore, the modified palisade technique described above can also be carried out under local anesthesia. This is especially useful for patients who are deemed unfit for a general anesthetic but will benefit from surgical repair of their perforation. As seen in the demographic data, the average age was 36 years, with a range of nine to 64 years. Also, many patients had comorbidities, including diabetes. Older patients and those with comorbidities are not good candidates for general anesthesia. Thus, having a technique that is doable under local anesthesia with good outcomes has a significant impact on practice. We do not believe the modified palisade technique will result in any increased pain or discomfort compared to other reported tympanoplasty procedures.

Studies have shown that graft uptake and success rate in tympanoplasty are associated with several factors, including size and site of the perforation, the surgeon’s level of expertise, age, gender, smoking, the middle ear mucosa state, and the presence of tympanosclerosis or cholesteatoma [25,26]. Pinar et al. stated that central perforations had better outcomes [26]. In addition, tympanosclerosis and large perforation size were associated with disrupted vascular supply and poor outcomes [25]. On the other hand, there are controversial studies showing that these factors do not influence the outcome of tympanoplasty [27]. In our study, there was no association between demographic data, comorbidities, smoking habit, site and size of perforation, and middle ear status. This makes the technique readily usable for almost all cases. However, not all patients who had cholesteatoma or tympanosclerosis had successful outcomes. There were a total of four cases, two of which had successful outcomes and two did not. One of them had a worsened air-bone gap, and in the other one, there was no graft uptake. Nevertheless, the number of cases is not enough to confirm this fact. Having more cases in the future will lead to more accurate results.

Onal et al. found that graft viability was significantly higher in patients whose ears remained dry for more than three months [27]. This fact is applicable to our technique, as all of our patients had a dry perforation for a minimum of three months. Conversely, Nagle et al. showed significant tympanoplasty success rates in dry ears (88%) and wet ears (74%) [28].

However, both results were statistically non-significant. Moreover, a study conducted in 2016 showed that there is little difference in the outcomes in dry vs. wet ears [29]. As for the etiology of the perforation, it could be infectious, iatrogenic (due to a grommet), or traumatic; it did not have any impact on the outcomes of surgery in our study, as well as in the study by Westerberg et al. [30].

**Figure 5:**
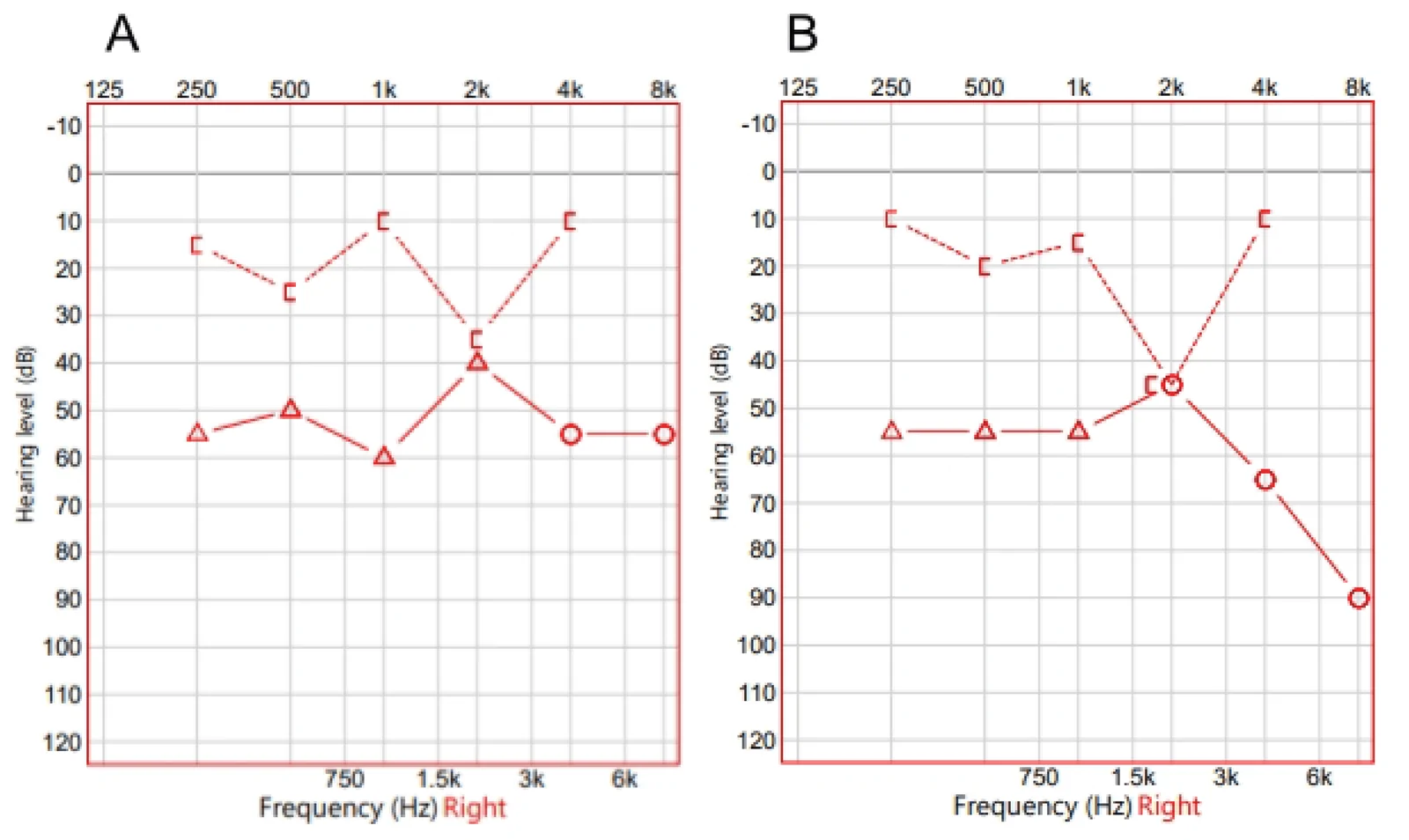
Pre- and postoperative audiometry with persistent air-bone gap. (A) Preoperative pure-tone audiometry. (B) Postoperative pure-tone audiometry.

The success of tympanoplasty is divided into functional and anatomical success. Functional success is defined as an air-bone gap ≤20 dB postoperatively, and anatomical success is having an intact graft in the dry ear [15]. Many studies showed that cartilage tympanoplasty had clinically and statistically significant functional success [31]. In our study, the functional success was 84.2%, and the anatomical success was 78.9%. The two patients who did not achieve functional success both had disrupted ossicular chains, and one of them had cholesteatoma as well. Figure 5 shows the pre- and postoperative audiometry, with an increased air-bone gap in the last-mentioned patient.

**Figure 6:**
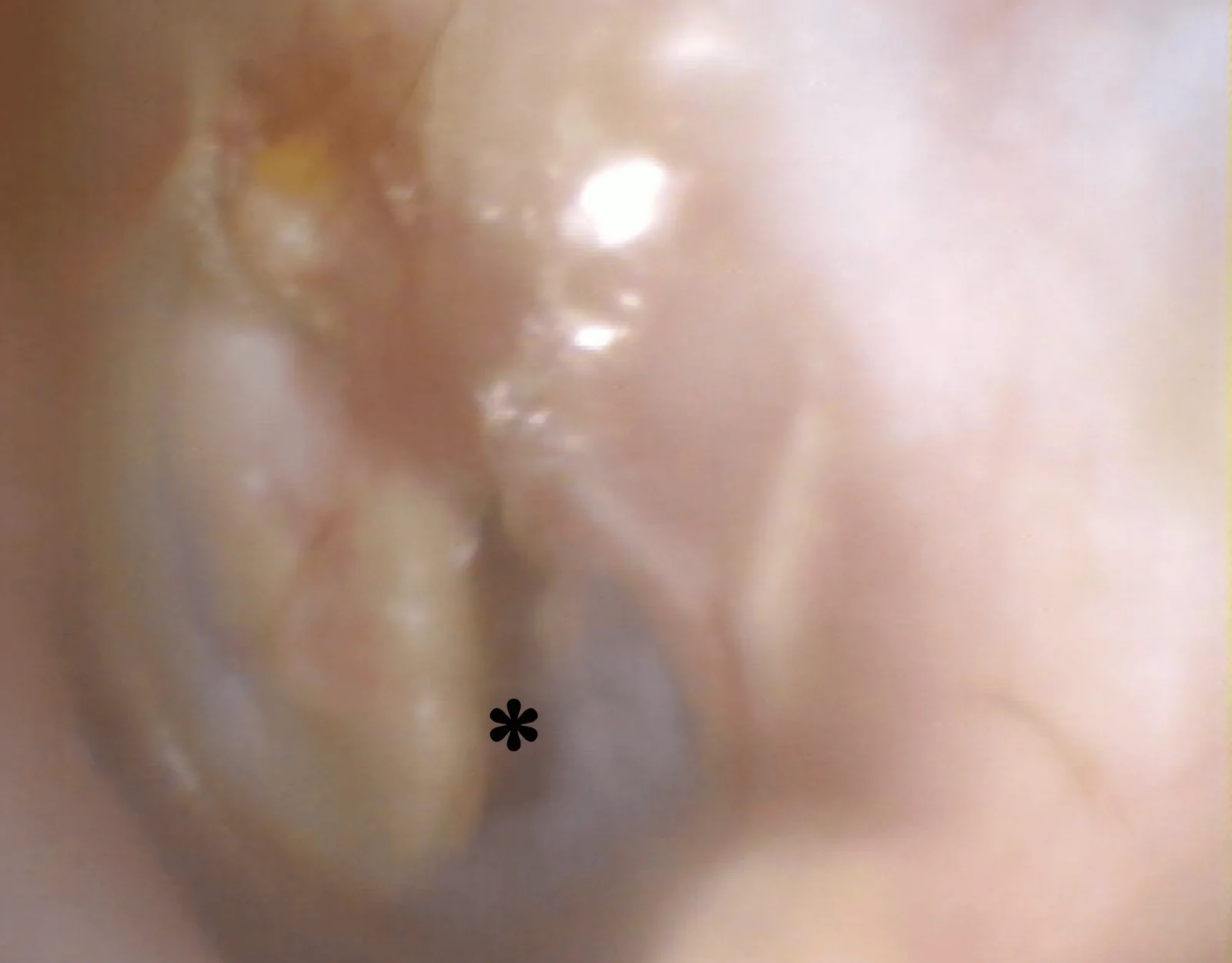
Posterior dry perforation of the left tympanic membrane. * Site of tympanic membrane perforation.

**Figure 7:**
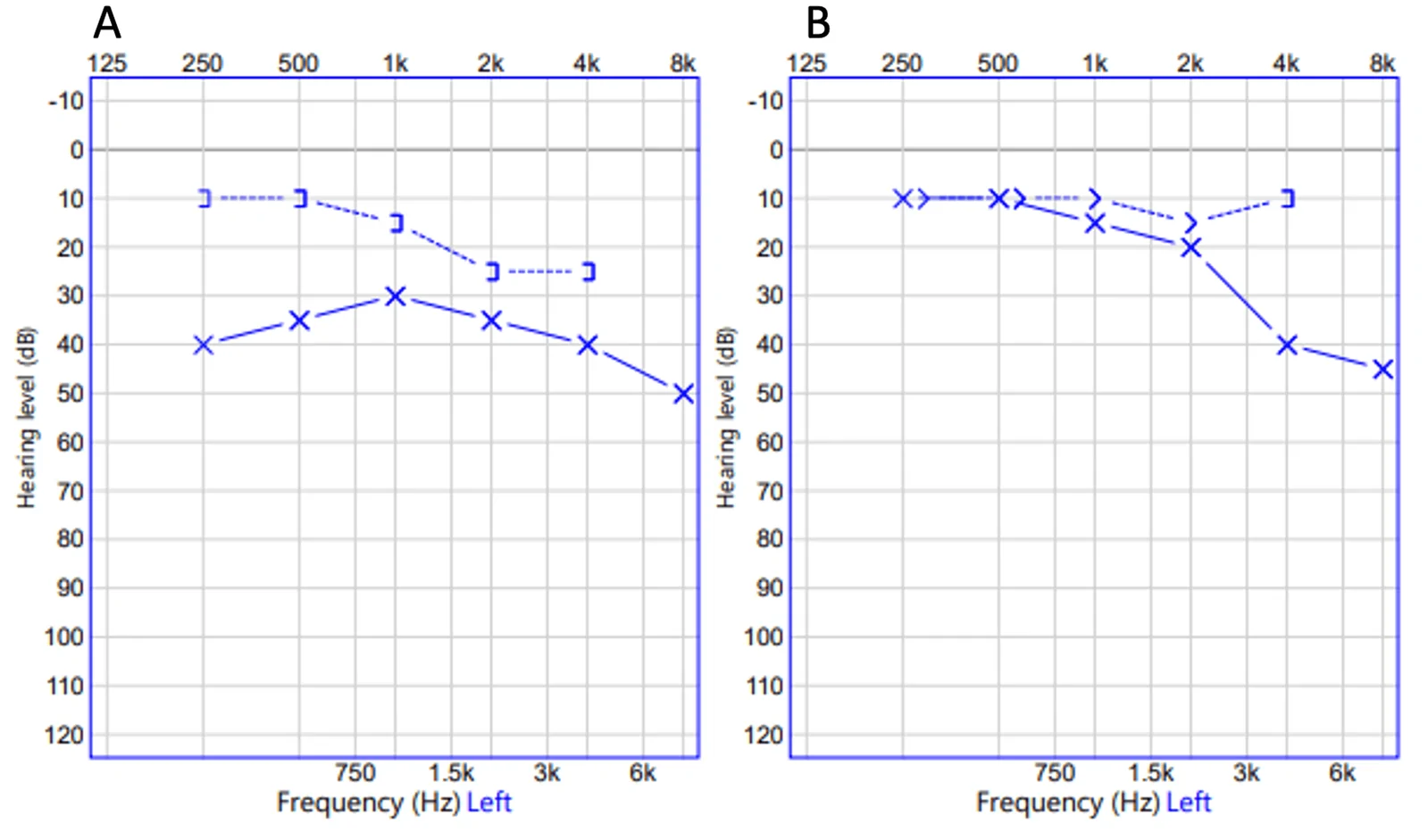
Pre- and postoperative audiometry with improvement in air-bone gap. (A) Preoperative pure-tone audiometry. (B) Postoperative pure-tone audiometry.

**Figure 8:**
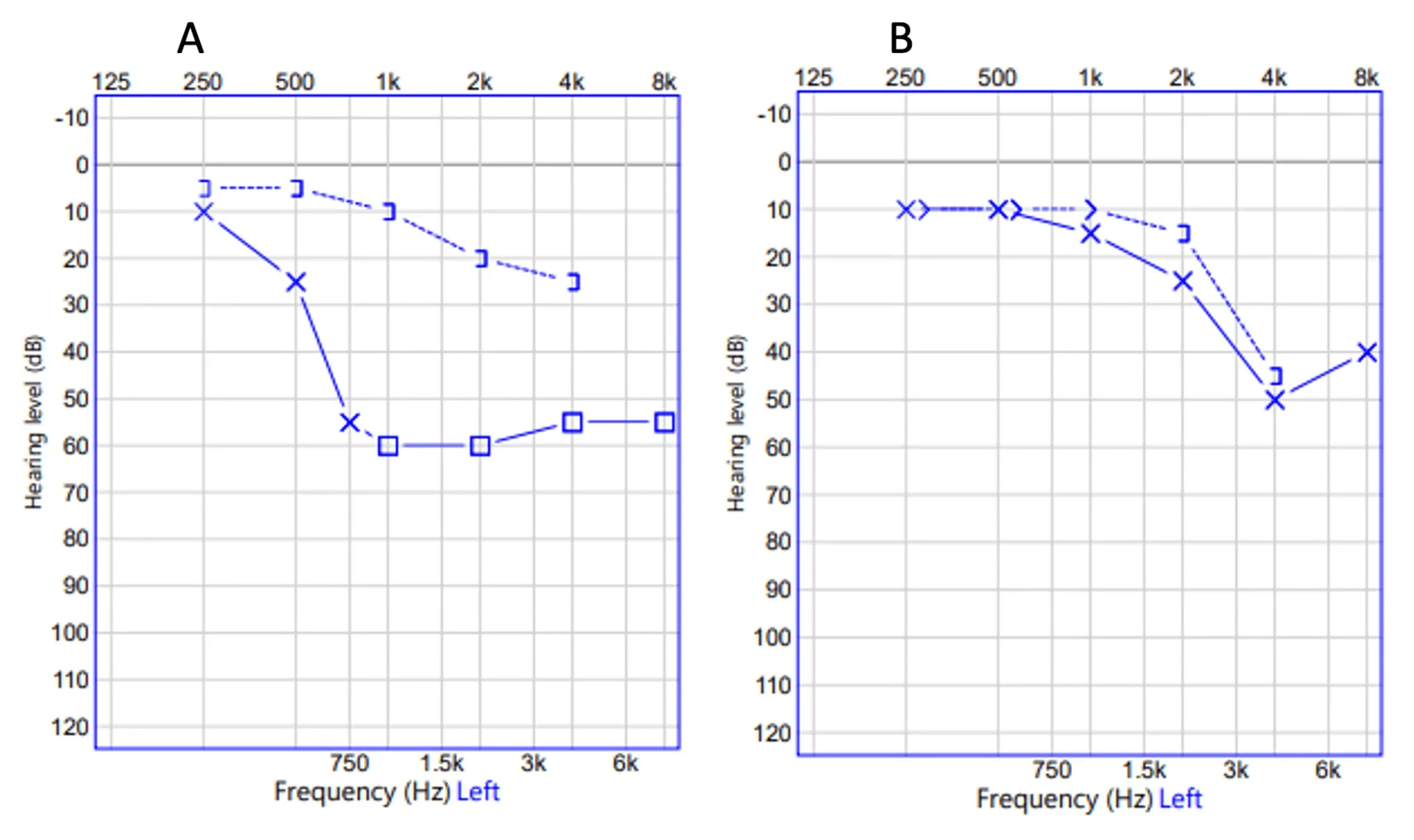
Pre- and postoperative audiometry with improvement in air-bone gap. (A) Preoperative pure-tone audiometry. (B) Postoperative pure-tone audiometry.

Of the four cases who did not achieve anatomical success, one of them underwent fat tympanoplasty in the clinic. The patient had complete graft uptake afterwards. Another patient, on the other hand, had persistent posterior perforation, medium in size and dry (Figure 6). The patient, however, is asymptomatic and satisfied with her outcomes. Figures 7, 8 show the air-bone gap improvement in these two cases.

Studies have shown that there is a debate regarding surgical outcomes in comparison with the transcanal and postauricular approaches. Rahman and Atikuzzaman found that the transcanal approach had better outcomes in terms of complications and hearing improvement, while graft uptake was better in the postauricular technique [32]. Huang et al. concluded that functional success was similar between both techniques [33]. On the other hand, Kappagantu and Gupta stated that the transcanal approach had fewer postoperative complications and pain, with better cosmetic outcomes [34]. However, there was no difference in terms of functional success between both approaches [34]. In our study, out of all cases, only three underwent the postauricular approach. All others were transcanal approaches.

These data are not sufficient to draw conclusions about the outcome difference between the two approaches. Having more cases in the future with an equal number of each approach will help in exploring this aspect.

Bayram et al. demonstrated that surgical experience is one of the factors that has an impact on the outcome of tympanoplasty [15]. Our study shows the opposite: some of the cases were performed by a consultant having more than 20 years of experience, and others were performed by senior residents, who had less than 10 years of experience. Despite that, all had successful outcomes. This indicates that the technique is simple enough to be used by all physicians at different levels of experience while maintaining excellent outcomes.

Limitations

Nevertheless, this technique requires meticulous surgical skills and will therefore lead to increased operating time. This is considered one of the challenges of our new technique. However, as this skill is practiced more regularly, we believe that this challenge can easily be overcome. In addition, there is an option of developing and using a cartilage slicer for improved accuracy, as designed by Parab et al. in 2018 [35].

Furthermore, there is an increased risk of disrupting the graft while carrying out a non-through-through excision of the longitudinal strips of the cartilaginous component of the graft. We believe this increased risk is negligible. Additionally, middle ear adhesions have been reported in composite grafts. This risk is applicable to our modified technique and is expected to have similar rates compared to other techniques that incorporate composite grafts in tympanoplasties [36].

Moreover, one of the limitations of our study is the small sample size. Conducting further studies with a large population will provide us with more detailed and accurate information.

## Conclusions

Composite graft tympanoplasties have grown in popularity over the last two decades to repair tympanic membrane perforations. In this study, we assess the success and efficacy of a modified palisade tympanoplasty. This new technique had significant functional and anatomical success, in terms of graft uptake and improvement of the air-bone gap in audiometry, pre- and postoperatively. The demographics, comorbidities, and perforation features had no impact on the successful outcome, with the exception of patients with tympanosclerosis or cholesteatoma. With repeated practice, this technique is a skill that we believe will be carried out by many surgeons for its applicability and simplicity once mastered.
